# Association of maternal anemia with other risk factors in occurrence of Great obstetrical syndromes at university clinics, Kinshasa, DR Congo

**DOI:** 10.1186/s12884-015-0623-z

**Published:** 2015-08-21

**Authors:** Barthélémy Tandu-Umba, Andy Muela Mbangama

**Affiliations:** Department of Obstetrics and Gynecology, University Clinics, University avenue Campus, PO Box 123, Kinshasa XI, DR Congo

## Abstract

**Background:**

Maternal anemia, a common situation in developing countries, provokes impairment of nutrients/oxygen supply to the placenta-fetus unit that leads to Great obstetrical syndromes (GOS). In our setting, however, occurrence of GOS has been found also depending on variables existing prior to pregnancy such as diabetes in family, hypertension in family, previous macrosomia, stillbirth, SGA and pre-eclampsia as well as overweight/obesity. Our study thus aimed to determine the magnitude of maternal anemia and its association with these pre-pregnancy high-risk variables in occurrence of GOS.

**Methods:**

This is a cross-sectional study including women delivered at the University Clinics of Kinshasa, DR Congo, 12. during 18 months. Anemia was stated at hemoglobin blood concentration < 10 g/dL. Sampled women were checked for pregnancy high-risk factors and pregnancy complications. Odds ratios (95 % confidence intervals) were calculated to establish associations of anemia with various variables. Multivariate calculations aimed to isolate variables influencing these associations. The p <0.05 was considered significant.

**Results:**

The study sample included 412 women, among whom 220 (53.4 %) were diagnosed anemic. Anemia was found significantly linked to malaria, urinary infection, cesarean section, prematurity, SGA and stillbirth whose risk was 1.6 – 6.1 times augmented. Anemia was also found linked to pre-pregnancy high-risk factors such as age < 18 and ≥ 35 years, previous miscarriage, grand multiparity, diabetes in family, previous prematurity, overweight/obesity, previous cesarean section and previous pre-eclampsia, all of them enhancing the link of maternal anemia with complications.

**Conclusion:**

Maternal anemia is very prevalent among pregnant women of our setting. It strongly contributes to worsening of morbidities that act with pregnancy high-risk factors in raising the risk of cesarean section, prematurity, SGA and stillbirth.

## Background

Perinatal morbidity and mortality are dominated by five common conditions (premature labor/delivery, premature rupture of membranes, small for gestational age, congenital anomalies and pregnancy induced‐hypertension) that belong to **«** Great Obstetrical Syndromes » (GOS) [1]. Their global importance has been growing because they are strongly linked to future non communicable diseases (NCDs) in offspring. Indeed, due to adaptive responses needed by fetus to face these pathological conditions, permanent changes are likely to occur in its lipid metabolism (low lipoproprotein cholesterol) [2], number of cells of the pancreas (underdeveloped pancreas) [3] and haemostatic factors (elevated concentrations of fibrinogen and factor VII) [4], which raise the risk of cardiovascular diseases (such as heart attacks and stroke), cancers and diabetes in adult life. These are 3 of the four main NCDs (the fourth being chronic respiratory diseases) that are projected to be the leading causes of global deaths by 2030 [5]. Almost 80 % of deaths owed to NCDs occur in low/middle-income countries [6]. Therefore, tackling NCDs by means of their ever known risk factors (malnutrition, sedentary lifestyle, exposure to cigarette’s smoke and alcohol abuse) [5, 6] needs to be accompanied by prevention/treatment of GOS.

Maternal anemia, a common situation in developing countries [7–9], provokes impairment of nutrients/oxygen supply to the placenta-fetus unit, an intra-uterine insult that leads to GOS [7]. In our setting, however, occurrence of premature labor/delivery, premature rupture of membranes, small for gestational age, congenital anomalies and pregnancy induced‐hypertension has been found also depending on variables existing prior to pregnancy such as diabetes in family, hypertension in family, previous macrosomia, stillbirth, SGA and pre-eclampsia as well as overweight/obesity [10]. Our study thus aimed to determine the magnitude of maternal anemia and its association with these pre-pregnancy high-risk variables in occurrence of GOS.

## Methods

This is a cross-sectional study including women delivered at the maternity of the Department of Obstetrics & Gynecology, University Clinics of Kinshasa, DR Congo, during 18 months (March 2012 throughout August 2014). The study protocol received ethical approval from both the Faculty of Medicine and the Department of Obstetrics & Gynecology Ethics Boards of the University Clinics of Kinshasa, and was conducted in accordance with the Helsinki Declaration. All participants provided informed verbal consent.

Every woman admitted for delivery was included if fulfilling our inclusion criteria: 1) singleton pregnancy and 2) having been followed for antenatal care in our clinics so that pregnancy complications could be recognized and registered. Women with debilitating pathologies or having been treated with a cervical cerclage and those known to have sickle-cell hemoglobin were excluded. Due to financial constraints laboratory assessment of blood, stool and urine during antenatal care may lack for several women. Sampled women were scheduled to have their hemoglobin status established upon entering the labor ward, irrespective of having previously benefited from iron supplement, de-hookworming and anti-malarial medications. Hemoglobin measurement was made from complete blood collected in an appropriate tube with citrated anticoagulant (EDTA – ethylenediamine-tetraacetic acid), thereafter processed using an automate machine (Awareness Technology Stat Fax® 4500 Chemistry analyzer) designed to measure other blood components. Gestational age ≥28 weeks to define delivery was based on the last menstrual period, an ultrasonography performed close to the first trimester and a birth weight ≥1000 g. Anemia is globally considered as the reduction in the hemoglobin blood concentration of the peripheral blood below 11-13 g/dL (depending on age, sex, altitude and pregnancy status) [7]. In this study, however, it was stated according to local references as hemoglobin bloodconcentration <10 g/dL [9]. Owing to lack of pre-pregnancy weight, overweight/obesity was defined as postpartum body mass index (BMI) ≥28 kg/m^2^ according to ranges previously defined in our setting [11].

Prematurity was considered at gestational age <37 weeks, as clinically estimated by a pediatrician. All women were checked for pre-pregnancy risk factors represented by socio-demographic and clinical variables: single status or not living with a partner, primiparity, grand multiparity (≥5), age <18 or ≥35 years, height <150 cm, personal medical/surgical morbidities, and mother/infant problems in previous pregnancies. Previous miscarriage was considered as at least two previous spontaneous abortions (consecutive or not). Maternal and perinatal complications related to just-terminated pregnancy were recorded, mostly those recognized as GOS: premature labor/delivery, premature rupture of membranes, small for gestational age (SGA), congenital anomalies and pregnancy induced‐hypertension. High arterial blood pressure (hypertension) was considered as ≥140/90 mmHg. Urinary tract infection was based on reported positive urine culture during the antepartum period. Neonatal distress was defined as an Apgar score <7 at the fifth minute. Low birth weight (LBW) and macrosomia were defined as birth weight < 2500 g and ≥4000 g, respectively. SGA was stated as an infant born with a birth weight less than the 10th centile according to local charts. Stillbirth was considered when a baby was born with an Apgar score = 0. Data were registered using Microsoft Excel (Microsoft Corporation, Redmond, WA, USA, 2007). After transfer to SPSS (version 18.0; SPSS Inc., Chicago, IL, USA) for statistical calculations, univariate analyses (odds ratios [ORs]) were stratified in a dichotomic way (having or not having anemia) to establish whether there was significant association with pre-pregnancy risk factors and pregnancy pathological situations. Multivariate calculations (regression) using various risk factors were aimed at isolating those significantly influencing occurrence of complications in anemic mothers. Results are given with 95 % confidence intervals (CIs), and p <0.05 was considered significant.

## Results

A total of 1934 pregnant women attended the antenatal clinic of our maternity and 751 women were delivered during the study period. Based on recruitment criteria the study sample included 412 women, whose general characteristics are presented in Table 1. The majority of them (272 = 66 %) were vaginally delivered (34 % by cesarean section). No maternal death was registered among sampled women. Socioeconomic status and premature rupture of membranes were discarded from the study due to inappropriate registration of items aimed at assessing them.

**Table 1:** General characteristics of the study group (n = 412)

Parameters	Mean ± SD
Age (yr)	30.5 ± 6.0
Weight (kg)	77.9 ± 1.1
Height (cm)	1.64 ± 0.1
BMI (post-partum) (Kg/m^2^)	28.5 ± 5.8
Haemoglobin (g/%)	9.9 ± 1.3
Gravidity	2.9 ± 1.8
Parity	2.5 ± 1.6
Gestational age	37.9 ± 2.1
Birth weight (gr)	2955.7 ± 575.4
Apgar score	7.3 ± 2.3

Anemia as hemoglobin blood concentration < 10 g/dL was diagnosed in 220 women (53.4 %). Among demographic and clinical risk factors presented in Table 2, the most prevalent were overweight/obesity (51.5 %), age ≥35 years (27.4 %), previous caesarean section (19.9 %), previous miscarriage (12.1 %) and grand multiparity (11.2 %). Pathological situations encountered during pregnancy are presented in Table 3. In unadjusted univariate analysis malaria, urinary tract infection, cesarean section, prematurity, SGA and stillbirth were significantly linked to anemia, having their risk 1.6–6.1 times augmented in anemic mothers (in bold in Table 3). Mother and infant’s adverse outcomes for which significant associations were found with maternal anemia were also significantly linked to pre-pregnancy risk factors such as age < 18 yrs, age ≥35 years, single status, previous miscarriage, grand multiparity, diabetes in family, previous prematurity, previous LBW, overweight/obesity, previous cesarean section and previous pre-eclampsia (Table 4). All these factors were next included in multivariate regression calculations in order to isolate those independently influencing occurrence of pregnancy complications in anemic mothers (Table 5). The link of maternal anemia with malaria was found significantly influenced by obesity, age < 18 yrs and diabetes in family. No factor was found linked to the association of anemia with urinary tract infection. Obesity significantly influenced the link of anemia with both cesarean section and prematurity. Single status and previous LBW significantly augmented the link of anemia with SGA while age ≥35 raised that of anemia with stillbirth.

**Table 2:** Distribution of pregnancy risk factors in the overall study group (n = 412)

Général charactéristics	Number	Percent
Age < 18 ans	8	1.9
Age ≥ 35 ans	113	27.4
Single status	44	10.7
Primiparity	140	34
Grand multiparity	46	11.2
BMI (postpartum) ≥ 28	212	51.5
Height < 150 (cm)	20	4.9
Previous and current morbidities		
Hypertension in family	28	6.8
Diabetes in family	15	3.6
Previous miscarriage	50	12.1
Current hypertensive disorders	27	6.6
Diabetes	4	1.0
Previous pre-eclampsia	18	4.4
Previous caesarean section	82	19.9
Previous prematurity	5	1.2
Previous LBW	24	5.8
Previous stillbirth	5	1.2
Previous acrosomia	26	6.3

**Table 3:** Association of maternal anemia with pregnancy complications (Unadjusted OR)

	Number	Percent	*p*-value	OR	CI	
Maternal complications						
Malaria	78	19	.000	2.4	1.6	3.8
HIV infection	1	.2	.5	1	.9	1
Urinary infection	62	15	.03	1.6	1.0	2.5
Threatened abortion	28	6.8	.4	1.2	.5	2.6
Cervicovaginitis	15	3.6	.09	2.3	.8	6.6
Tuberculosis	1	.2	.5	1	.9	1
Intestinal amibiasis	5	1.2	.5	1.3	.2	7.9
Placental abruption	2	.5	.7	1.1	.7	18.2
Hypertensive disorders	27	6.6	.51	1.1	.41	3.1
Gestational diabetes	2	.5	.7	1.1	.7	18.2
Cesarean section	140	34	.000	6.1	3.8	9.8
Perinatal complications						
Prematurity	56	13.6	.01	2	1.1	3.7
Neonatal distress	50	12.1	.2	1.4	.7	2.5
SGA	80	19.4	.01	1.7	1.8	2.3
Macrosomia	22	5.3	.2	1.6	.6	3.8
Stillbirth	30	7.3	.001	3.8	1.7	8.9
Congenital malformation	9	2.2	.4	1.4	.4	5.3

**Table 4:** Association of maternal pre-pregnancy risk factors with pregnancy outcomes in anemic mothers (Unadjusted OR)

Risk factors	Outcomes	p	OR	CI	
Overweight/Obesity	Malaria	.032	1.7	1.0	2.7
Age < 18 years		.045	4.5	1.1	18.2
Diabetes in family		.012	4.01	1.4	11.4
Previous prematurity		.049	6.6	1.1	40.4
Previous miscarriage	Urinary infection	.020	1.9	1.0	3.5
Overweight/Obesity	Cesarean section	.041	4.6	1.3	8.5
Previous PE		.001	4.9	1.9	13.1
Overweight/Obesity	Prematurity	.035	4.2	1.8	7.9
Previous miscarriage		.005	2.5	1.2	5.1
Age ≥ 35 years	Stillbirth	.002	3.4	1.6	7.1
Grand multiparity		.001	4.8	2.1	11.1
Previous prematurity		.045	9.0	1.4	56.2
Single status	SGA	.027	2.1	1.1	4.2
Previous LBW		.000	4.7	2.0	10.9

**Table 5:** Association of maternal anemia with pregnancy complications adjusted to risk factors (Adjusted OR)

Adjustment factors	Outcomes	p	OR	CI	
Anemia	Malaria	.000	2.4	1.6	3.8
Obesity		.04	1.7	1.0	3.0
Age < 18 yrs		.03	5.3	12.2	22.7
Diabetes in family		.008	4.4	1.5	13.0
Previous prematurity		.06	5.8	.9	36.8
Anemia	Urinary infection	.03	1.6	1.0	2.5
Previous miscarriage		.02	.4	.2	.9
Anemia	Cesarean section	.000	6.1	3.8	9.8
Obesity		.04	4.6	1.3	8.5
Previous PE		.001	4.9	1.9	13.1
Anemia	Prematurity	.03	2	1.1	3.6
Obesity		.02	2.1	1.1	3.8
Previous miscarriage		.02	.4	.2	.8
Anemia	Stillbirth	.001	3.8	1.7	8.6
Age ≥35 years		.002	3.4	1.6	7.1
Anemia	SGA	.1	1.5	9	2.5
Single status		.01	2.5	1.2	5.1
Previous LBW		.000	5.3	2.3	12.5

## Discussion

This study aimed to determine the magnitude of anemia in pregnant women of the study setting and its association with other high-risk factors in occurrence of great obstetrical syndromes. In our study hemoglobin check was made in labor ward, meaning that patients might have benefited from corrective measures during pregnancy. Therefore, the mean hemoglobin blood concentration of the sample (9.9 ± 1.3 g/dl) reflects the severity of anemic situation among pregnant women of the study setting.

Although the study did not report any lethal complication, fear exists that this situation might precipitate maternal death postpartum. The current rate of anemic pregnant women (53.4 %) is similar to that of fifty years ago in the same setting and in the range of rates reported in 2003 in Lubumbashi (50–80 %), South Eastern DR Congo [9]. Such an ongoing situation in the country is probably owed to the same main causes reported in developing/poor countries: undernutrition, blood/intestinal parasitoses, urinary tract and systemic chronic infections and absence of iron supplementation [9, 12, 13]. In our series associations found between maternal anemia and malaria and urinary tract infection are in accordance with previous literature pointing out that the anemic condition might be caused or worsened by these morbidities [14]. They still represent a major target in strategies for prevention/reduction of pregnancy complications related to maternal anemia. Since iron-folic acid supplementation, de-hookworming medication and anti malarial prevention/treatment represent a package that has proven to reduce maternal anemia in developing areas [15, 16], we postulate that its implementation in our setting might have been insufficient, probably requiring a money-free supply.

As of other maternal complications, including hypertensive disorder that belongs to GOS, none was significantly associated with anemia. We know of no study having demonstrated such links, although maternal folic acid deficiency is cited among causal factors of placental abruption. Anemia is also known to induce a placental ischemia, one of processes involved in initiation of premature labor [7].

In offspring we found significant links of maternal anemia with prematurity, SGA and stillbirth, corroborating previous literature [5–9, 17]. The link with congenital anomalies was not significant. These prominent perinatal outcomes can be explained by malaria and urinary tract infection as well. Both anemia and malarial parasitemia have been postulated to dysregulate placentation and angiogenesis [17], and this may lead to pathological changes in both uterine and umbilical artery blood flow [17] (resulting in intrauterine growth retardation) as well as to preterm onset of labor. As such, premature labor is assumed to represent a late clinical manifestation of a long lasting pathophysiological situation. According to Romero et al [18], although a few women might benefit from tocolysis, uterine contractions in this case should therefore be left to proceed until (premature) delivery. Urinary tract infection in pregnancy is known to increase the risk of pyelonephritis, amnionitis, premature labor, low birth weight and perinatal death [19, 20]. Mazor-Dray et al. [21] demonstrated that it is independently associated with pre-term delivery, pre eclampsia and restricted fetal growth. The role of malaria and urinary tract infection is also expected to increase fetal complications that lead to cesarean section. Malhotra et al [22] also reported severe anemia to be associated with increased operative delivery. This could explain the link we found between anemic situation and cesarean section. The latter, in turn, is in odds to be complicated by anemic situation, mostly intra- and postpartum.

Our results also showed significant links between maternal anemia and pre-pregnancy risk factors such as age < 18 or ≥35 years, single status, previous miscarriage, grand multiparity, diabetes in family, previous prematurity, overweight/obesity, previous cesarean section and previous pre-eclampsia. These variables are well known to put pregnancy at risk of maternal and perinatal adverse outcomes by potentiating co-morbidities such as preeclampsia and urinary tract infection that, in turn, worsen the risk of fetal growth restriction, preterm birth and stillbirth [18, 23]. However, only diabetes in family, overweight/obesity and previous pre-eclampsia, some of major risk factors of gestational diabetes, were found influencing the occurrence of GOS. We thus postulate that these pre-pregnancy risk factors are likely to represent a proxy for a baseline sensitivity, probably owed to genetic, in occurrence of GOS.

No factor was found influencing the link of anemia with urinary infection. They thus act independently. The high rate of overweight/obesity seems to be in discordance with expected poor nutrition, which raises the question of additional tools to measure maternal nutrition, an issue not addressed in our study. Obesity significantly influenced the link of anemia with both cesarean section and prematurity. Either direct or not, the causative nature of these links is not completely understood although overweight/obesity has been shown to increase the likelihood of gestational diabetes which raises the risk of macrosomia and obstructed labor. In their study dealing with relationships between systemic and localized maternal co-morbidities with preterm birth, Auger et al [19] identified preeclampsia and anemia as the most important systemic maternal co-morbidities contributing to premature rupture of membranes. The latter leads to premature delivery as well as mother/infant’s infection.

Iron supplementation studies that have shown actual decrease in rates of LBW failed to eradicate adverse outcomes [24]. This raises the need of extending research to additional factors that might be potential determinants of both maternal hemoglobin concentration and pregnancy outcomes. Our findings support that variables existing prior to pregnancy range among those additional factors, mostly risk factors of gestational diabetes. In order to make current strategies more cost-effective in reducing the present and future burden of maternal anemic situation they should base the determination of women in actual need of money-free corrective measures. However, initiation of interventions targeting non modifiable factors prior to or early in pregnancy remains very difficult where antenatal care attendance is late.

### Strengths of our study

These last ten years most of literature addressing maternal anemia has been dominated by review articles and trials assessing the effect of iron use on maternal anemia, or emphasizing the part of blood and intestinal parasites into maternal anemic situation. So, there have been less research articles addressing potential action of other variables, including pre-pregnancy risk factors, that need to be accounted for in efforts to prevent or attenuate the maternal anemic situation.

### Weaknesses of the study

We can deplore that pathological situations encountered during pregnancy were to be recorded retrospectively from patients’ files. This excludes asymptomatic urinary tract, genital and certain malarial infections, cervical incompetence along with many other complications, provided these were not systematically searched for. So, actual rates of pregnancy complications might have been minored.

## Conclusions

Maternal anemia is very prevalent among pregnant women of our setting. It represents an important fetal insult that raises the risk of prematurity, SGA and stillbirth. This risk is steadily higher when maternal anemia is associated with risk factors of gestational diabetes. The latter might thus represent a pre-pregnancy condition likely 197. to serve as a proxy of baseline individual sensitivity. This is to be taken in account in order to prevent future NCDs 198. in both mothers and offspring.
